# The genome sequence of *Gardnerycteris keenani *(Chiroptera, Phyllostomidae, Stenodermatinae; Handley, 1960)

**DOI:** 10.12688/wellcomeopenres.26494.1

**Published:** 2026-08-18

**Authors:** Nancy B. Simmons, Melissa R. de Waal, Meike Mai, Larry N. Singh, Philge Philip, Laramie L. Lindsey, Ning Zhang, Jonathan L. Gray, Samuel C. Talbot, Brian P. O'Toole, Kirsty McCaffrey, Giulio Formenti, Linelle Abueg, Bonhwang Koo, Myrtani Pieri, Emma C. Teeling, Erich D. Jarvis, Sonja C. Vernes, Bat1K Consortium

**Affiliations:** 1 Department of Mammalogy, Division of Vertebrate Zoology, American Museum of Natural History, New York NY 10024, New York, 10024, USA; 2 Fairleigh Dickenson University, Madison, NJ, USA; 3 School of Biology, University of St Andrews, St Andrews, UK; 4 Paratus Sciences, New York, USA; 5 Center for Quantitative Life Sciences, Oregon State University, Corvallis, Corvallis, USA; 6 Vertebrate Genome Lab, The Rockefeller University, New York, USA; 7 Department of Life Sciences, School of Life and Health Sciences, University of Nicosia, Nicosia, Cyprus; 8 School of Biology and Environmental Science, University College Dublin, Dublin, Ireland

**Keywords:** Gardnerycteris keenani, genome sequence, chromosomal, Bat1K

## Abstract

We present a genome assembly from an individual male *Gardnerycteris keenani* (Chordata; Mammalia; Chiroptera; Phyllostomidae). The genome sequence is 2.2 Gb in span. The majority of the assembly is scaffolded into 32 chromosomal pseudomolecules, with the X and Y sex chromosomes assembled.

## Species taxonomy

Eukaryota; Metazoa; Chordata; Craniata; Vertebrata; Euteleostomi; Mammalia; Eutheria; Laurasiatheria; Chiroptera; Yangochiroptera; Phyllostomidae; Phyllostominae; Phyllostomini *Gardnerycteris*; *Gardnerycteris keenani* ( Baker et al., 2016; Cirranello et al., 2016; Simmons and Cirranello, 2025).

## Introduction

**Figure 1.**
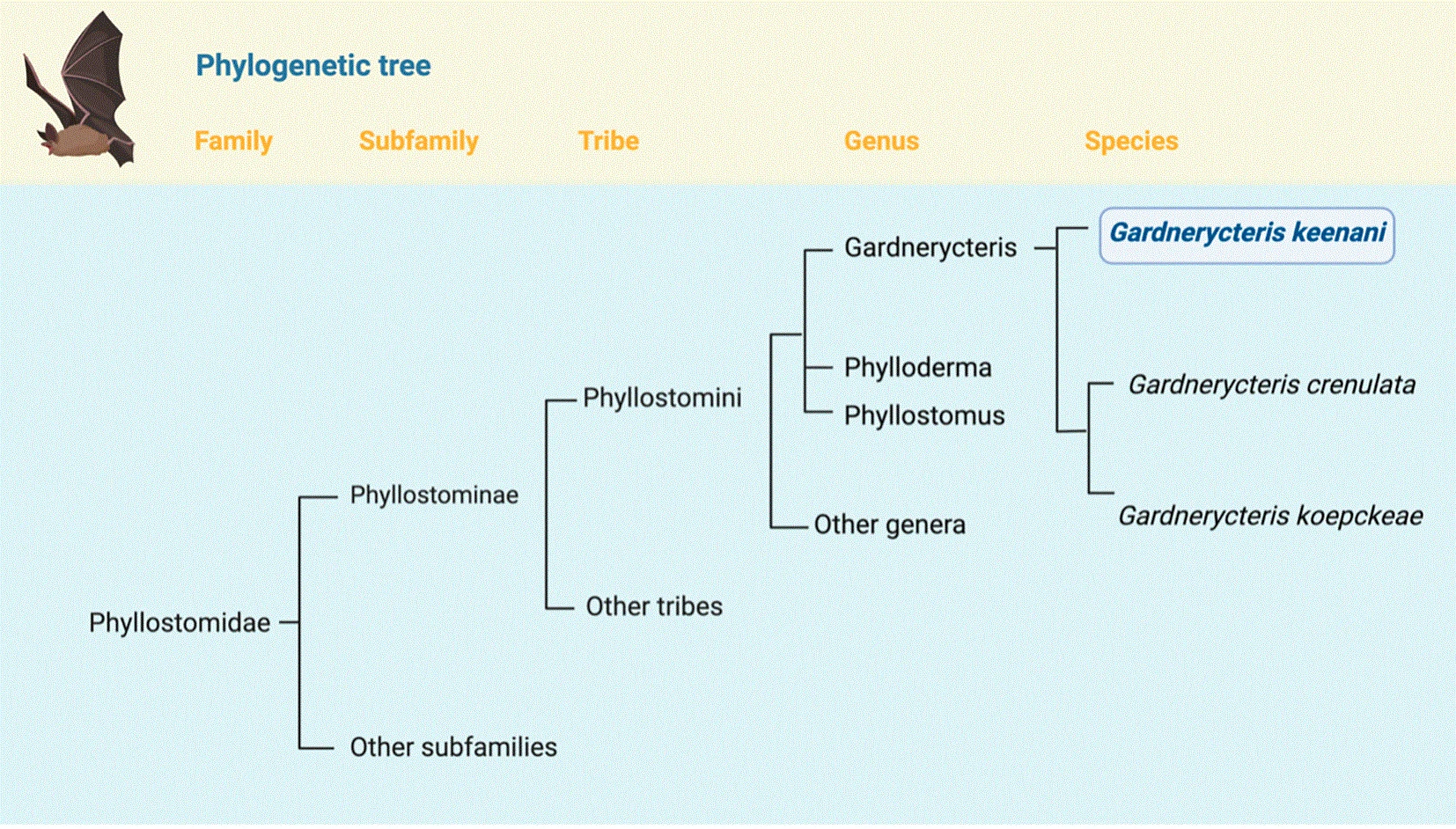
Position of *Gardnerycteris keenani* in the phylogeny of Family Phyllostomidae. *Gardnerycteris keenani* is one of 3 species currently recognized in the genus *Gardnerycteris* ( Hurtado and D’Elia, 2018; Simmons and Cirranello, 2025). *Gardnerycteris* belongs to the Tribe Phyllostomini, which currently includes 5 genera and 19 species ( Simmons and Cirranello, 2025). Phylogeny based on Hurtado and D’Elia (2018).

*Gardnerycteris keenani* ( Handley, 1960) is one of three species now recognized in the genus *Gardnerycteris*, a clade of medium-sized phyllostomine bats that is widespread across the Neotropics ( Baker et al., 2016; Cirranello et al., 2016; Simmons and Cirranello, 2025). These bats were previously placed in the genus *Mimon,* but phylogenetic analyses indicated that *Mimon* as thus constituted was polyphyletic, and Hurtado and Pacheco (2014) created a new genus for these taxa. *Gardnerycteris keenani* was considered a subspecies of *G. crenulata* (previously *Mimon crenulatum*) for many years (e.g., Koopman, 1994; Simmons, 2005; Williams and Genoways, 2007) but Hurtado and D’Elia (2018) demonstrated that it represents a distinct species ( Figure 1). As now defined, *Gardnerycteris keenani* occurs in Mexico, Belize, Guatemala, Honduras, Nicaragua, Costa Rica, Panama, Colombia, W Venezuela, W Ecuador, and W Peru ( Mammal Diversity Database, 2026; Simmons and Cirranello, 2025). In Central America, these bats are widespread but locally rare ( Reid, 2009).

*Gardnerycteris keenani* is a medium-sized phyllostomine (forearm = 46–55 mm, 10–18 g; Reid, 2009). Like other members of the genus *Gardneryceris*, *G. keenani* is characterized by distinctive external traits including a long, broad noseleaf that has crenulated margins; blackish dorsal fur; paler yellowish ventral fur with a dark base; ears that are large, pointed, and bicolored with a pink base and blackish tip; wing membranes that attach at the side of the foot; and a long tail and uropatagium ( Williams and Genoways, 2007; Reid, 2009). At the species level, *G. keenani* is distinguished from congeners by a combination of features including a broad, yellowish dorsal stripe; short and dense dorsal fur; presence of large hairs on the tip and short hairs on the base of the noseleaf; skull with relatively low sagittal crest; basioccipital breadth across cochleae relatively large; deep basisphenoid pits; and no vertical groove between the protocone and hypocone on the lingual cingulum of M1 and M2 ( Williams and Genoways, 2007; Hurtado and D’Elia, 2018).

*Gardnerycteris keenani* is found in evergreen and semideciduous forest, and sometimes in plantations and clearings near forested areas ( Reid, 2009). In Belize, we have captured them along trails cut through old-growth secondary forest (NBS and MDW, pers. observation). Little is known about the ecology of *G. keenani.* They apparently roost small groups clustered together in rotting logs, hollow tree stumps, and occasionally in buildings ( Reid, 2009). They are thought to be gleaners, with a diet consisting primarily of beetles with some hemipterans, flies, moths, whipscorpions, and occasionally small lizards ( Whitaker and Findley, 1980; Humphrey et al., 1983; Ingala et al. 2021).

*Gardnerycteris keenani* has not been assessed by the IUCN Redlist of Threatened Species because it was treated as conspecific with *Gardnerycteris crenulatum (= G. crenulata)* in the last assessment ( Solari, 2019).

*Gardnerycteris keenani* is known to be a host at least two species of nycteriibeid batflies in the genus *Basilia* ( Pastrana-Montiel et al., 2019; Wolff et al., 2023) as well as unique hemoplasma bacteria ( Volokhov et al., 2023).

## Genome sequence report

The genome was sequenced from a single male *Gardnerycteris* (field number BZ-1272, catalog number AMNH:Mammalogy:281952 collected near Lamanai Outpost Lodge, Indian Church Village, Orange Walk District, Belize, on 29 April 2022. A total of 20.7-fold coverage in Pacific Biosciences Hi-Fi long reads (contig N50 58.8 Mb) was generated after removal of all reads shorter than 10 kb. Primary assembly contigs were scaffolded with chromosome confirmation Hi-C data. The final assembly has a total length of 2.2 Gb in 187 sequence scaffolds with a scaffold N50 of 171 Mb ( Table 1). The majority, 99.2%, of the assembly sequence was assigned to 17 chromosomal-level scaffolds, representing 15 autosomes (numbered by sequence length, and the X + Y sex chromosomes. Chromosomal pseudomolecules in the genome assembly of *Gardnerycteris keenani* are shown in Table 2. The assembly has a BUSCO ( Simão et al, 2015) completeness of 98.0% using the vertebrata odb10 reference set. The assembly is fully phased, and both haplotypes are deposited therein.

**Table 1.** Genome data for *Gardnerycteris keenani.*

***Project accession data***	
Assembly identifier	mGarKee1.hap1
Species	***Gardnerycteris keenani***
Specimen	muscle, liver, heart, testes, spleen, kidney
NCBI taxonomy ID	**2358194**
BioProject	Bat1K: Accession: PRJNA489245; ID: 489245
BioSample ID	SAMN48711179
Isolate information	male
***Raw data accessions***	
Pacific Biosciences SEQUEL II	SRR34021279, SRR34021277, SRR34021275, SRR34021273 (.bam) SRR34021280, SRR34021278, SRR34021276, SRR34021274 (.fastq)
Hi-C Illumina	SRR34021282, SRR34021281
Assembly accession	GCA_050655435.1
Accession of Alternative haplotype	GCA_050655445.1
Span (Gb)	2.2
Number of contigs	290
Contig N50 length (Mb)	58.4
Number of scaffolds	187
Scaffold N50 length (Mb)	171.2
Longest scaffold (Mb)	221.2

* ***Gardnerycteris keenani*** BUSCO scores based on vertebrata_odb10 BUSCO set v5.3.2. C = complete [S = single copy, D = duplicated], F = fragmented, M = missing, n = number of orthologues in comparison.

**Table 2.** Chromosomal pseudomolecules in the genome assembly of *Gardnerycteris keenani.* ENA accession Chromosome Size (Mb) GC%. The chromosome number of ***Gardnerycteris keenani*** is 2n = ***32***.

GenBank accession	Chromosome	Size (Mb)	GC%
CM115949.1	1	221.25	41
CM115950.1	2	220.33	40.5
CM115951.1	3	217.67	41.5
CM115952.1	4	213.44	40
CM115953.1	5	177.95	42.5
CM115954.1	6	171.19	42.5
CM115955.1	7	143.06	41.5
CM115956.1	8	114.71	45.5
CM115957.1	9	108.19	43
CM115964.1	X	105.60	39.5
CM115958.1	10	95.66	40
CM115959.1	11	95.25	41.5
CM115960.1	12	78.20	47.5
CM115961.1	13	74.96	48
CM115962.1	14	61.80	43.5
CM115963.1	15	53.40	46.5
CM115965.1	Y	13.71	43.5

## Methods

The *Gardnerycteris keenani* specimen was a male individual collected on an American Museum of Natural History (AMNH) field expedition near Lamanai Archaeological Reserve in the Orange Walk District of Belize. The individual sampled was identified as *G. keenani* based on morphological traits (e.g., noseleaf structure, fur color and stiping) as described above. The bat was caught in a ground-level mist net set across a trail (“Rice Trail”) through old secondary forest near Lamanai Outpost Lodge (17.74692 N, 88.65652 W). All efforts were made to minimize any distress or suffering by the animal. The individual sampled was subjected to minimal handling after capture, and it was held in a clean cloth bag after capture as per best practices for field containment of bats. After species identification, the individual was euthanized humanely the same night it was captured. The animal was euthanized by isoflurane inhalation (<1 ml to moisten cotton ball, formula CHF _2_OCClHCF _3_, CAS number 26675–46-7; Manufacturer Piramal Critical Care, Supplier US Pharmacy Systems, Product code 5034-1FL-SOL-ORA), an approved and humane euthanasia method that rapidly causes unconsciousness within seconds and death within a minute or two with no significant suffering by the animal. Capture and sampling were conducted under Belize Forest Department Permit FD/WL/1/21(16) and samples were exported under Belize Forest Department permit FD/WL/7/22(07). All work was conducted with approval by the AMNH Institutional Animal Care and Use Committee (AMNHIACUC-20210614). All data were recorded and reported in accordance with the ARRIVE guidelines – see data availability section and Table 1. Tissues were removed from the subject individual immediately following euthanasia and were flash-frozen in a liquid nitrogen dry shipper, with the cold chain maintained from field to museum to laboratory.

DNA was extracted using Nanobind extraction from liver tissue following the Circulomics Nanobind HMW DNA Extraction Protocol. Pacific Biosciences HiFi libraries were constructed according to the manufacturer’s instructions. Hi-C data was generated using the Arima Hi-C+ High Coverage kit from the same tissue sample. Sequencing was performed by the Genomic Operations DNA Pipelines at Paratus Sciences on Pacific Biosciences Sequel IIe (HiFi reads) and Illumina NextSeq 2000 (Hi-C) instruments.

**Figure 2.**
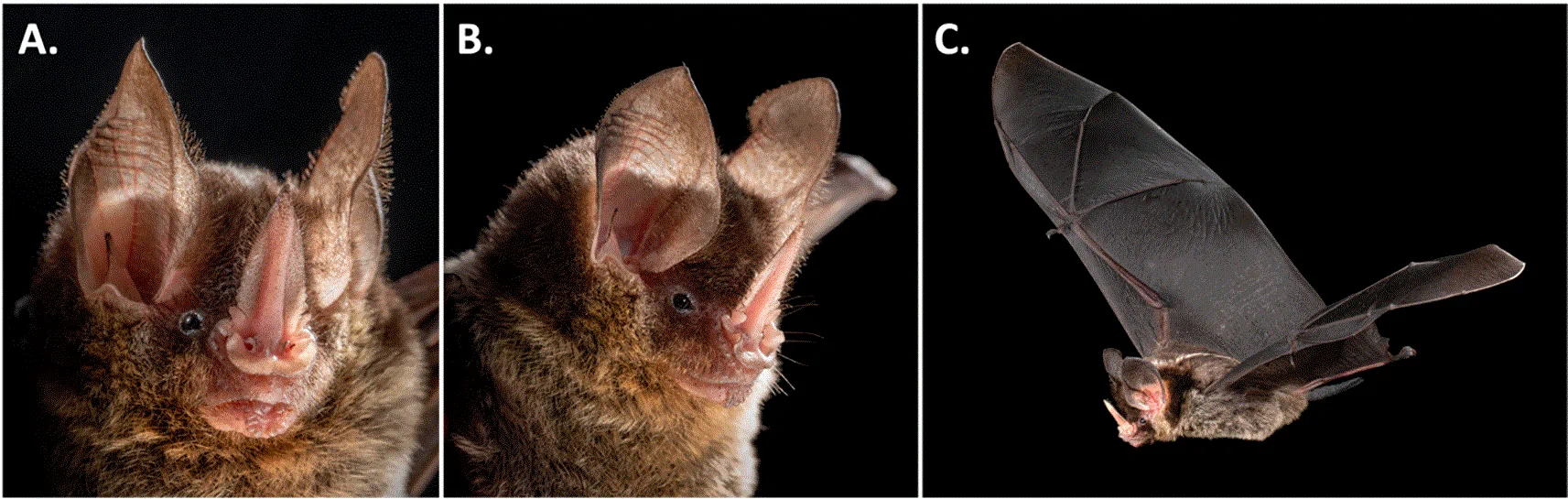
*Gardnerycteris keenani* Adult individuals of *Gardnerycteris keenani.* A and B, portraits showing ear, face, chin, and noseleaf morphology. C, *Gardnerycteris keenani* in flight. [Photos taken at Lamanai, Belize by Price Sewell (A, B) and by Brock and Sherri Fenton (C)].

Assembly was carried out following the Vertebrate Genome Project pipeline v2.0 ( Rhie et al., 2020) with a few modifications as follows. An initial QC analysis was performed on the raw BAM file using FastQC ( https://www.bioinformatics.babraham.ac.uk/projects/fastqc/). BAM files were converted to fastq format and merged for downstream processing. Genome size was estimated using GenomeScope2 ( Vurture et al., 2017). Hifiasm was used for genome assembly ( Cheng et al., 2021). The quality of the assembly was evaluated using Merqury ( Rhie et al., 2020) and BUSCO ( Manni et al., 2021). Scaffolding with Hi-C data ( Ghurye et al., 2019) was carried out with YaHs ( Zhou et al., 2023). PretextView was implemented to generate Hi-C contact maps. Figure 2– Figure 6 were generated using BlobToolKit ( Challis et al., 2020). Software utilised for the *Gardnerycteris keenani* analysis are depicted in Table 3.

**Figure 3.**
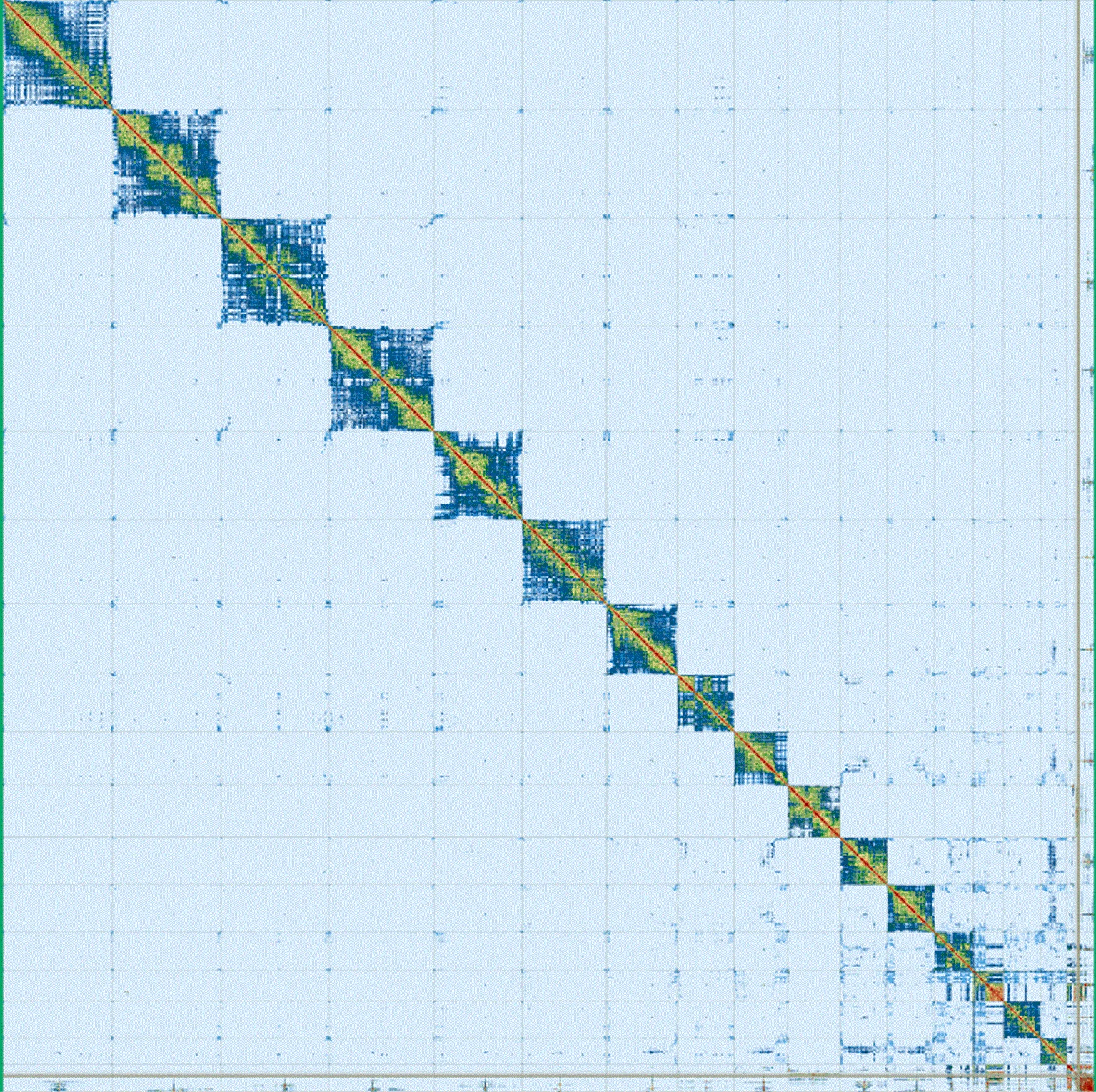
Hi-C Contact Map of the *Gardnerycteris keenani* assembly with 17 chromosomes, visualized using PretextView.

**Figure 4.**
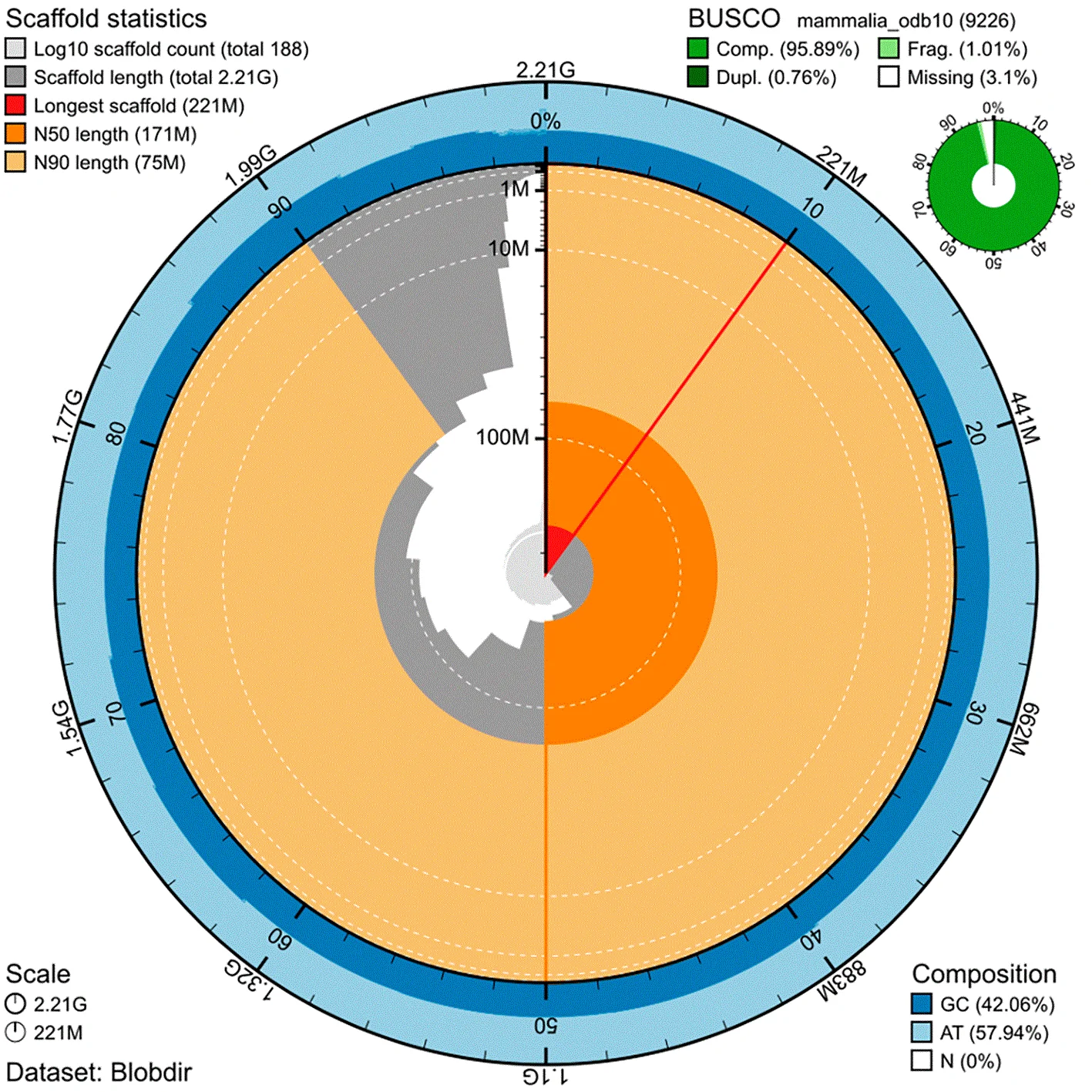
Genome assembly metrics generated using BlobToolKit for the *Gardnerycteris keenani* genome assembly. The larger snail plot depicts scaffold statistics including N50 length (bright orange) and base composition (blue). The smaller plot shows BUSCO completeness in green.

**Figure 5.**
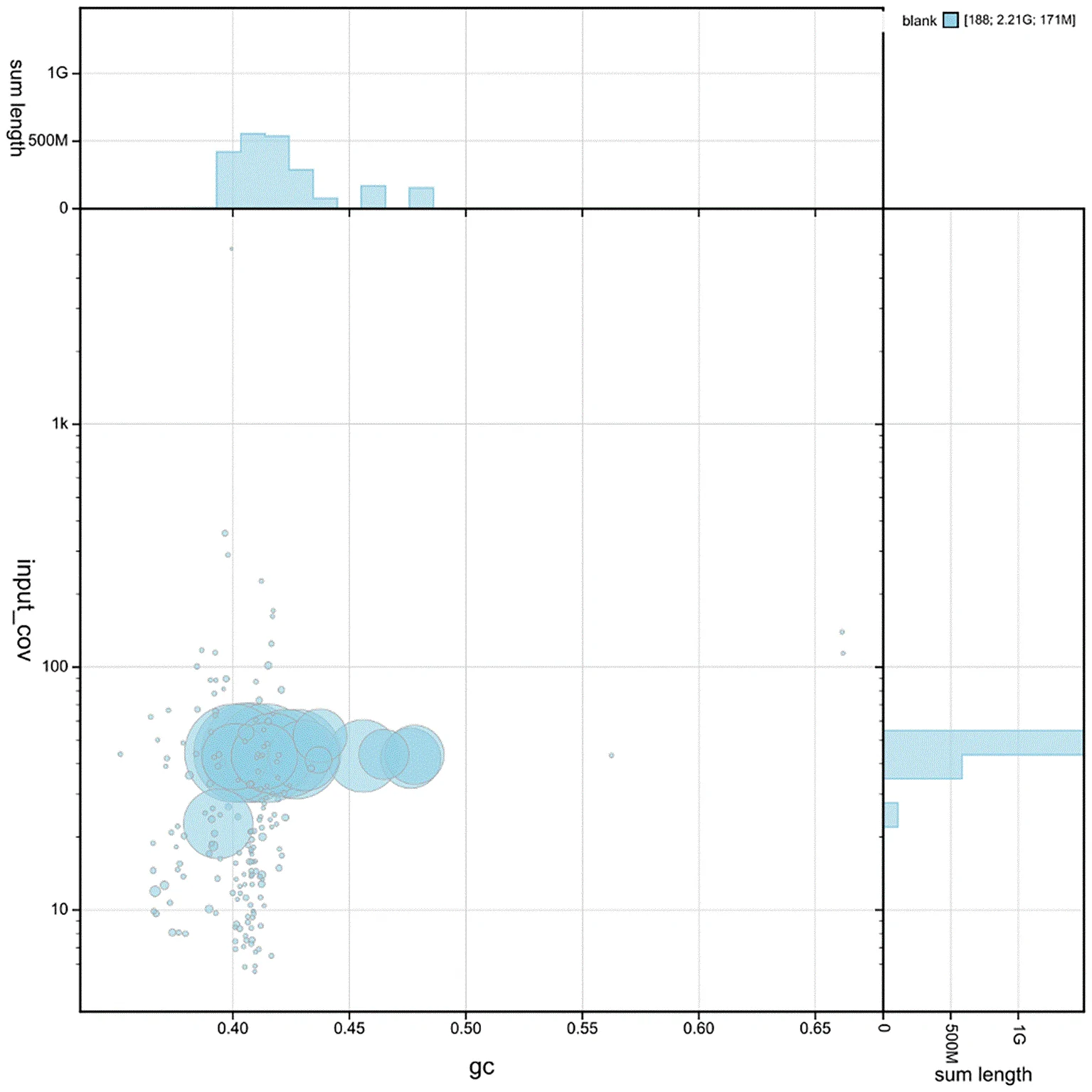
GC coverage plot generated for the *Gardnerycteris keenani* assembly using BlobToolKit. Individual chromosomes and scaffolds are represented by each circle. The circles are sized in proportion to chromosome/scaffold length. Histograms show the sum length of chromosome/scaffold size along each axis. Color of circles indicate taxonomic hits of each Phylum represented in the assembly.

**Figure 6.**
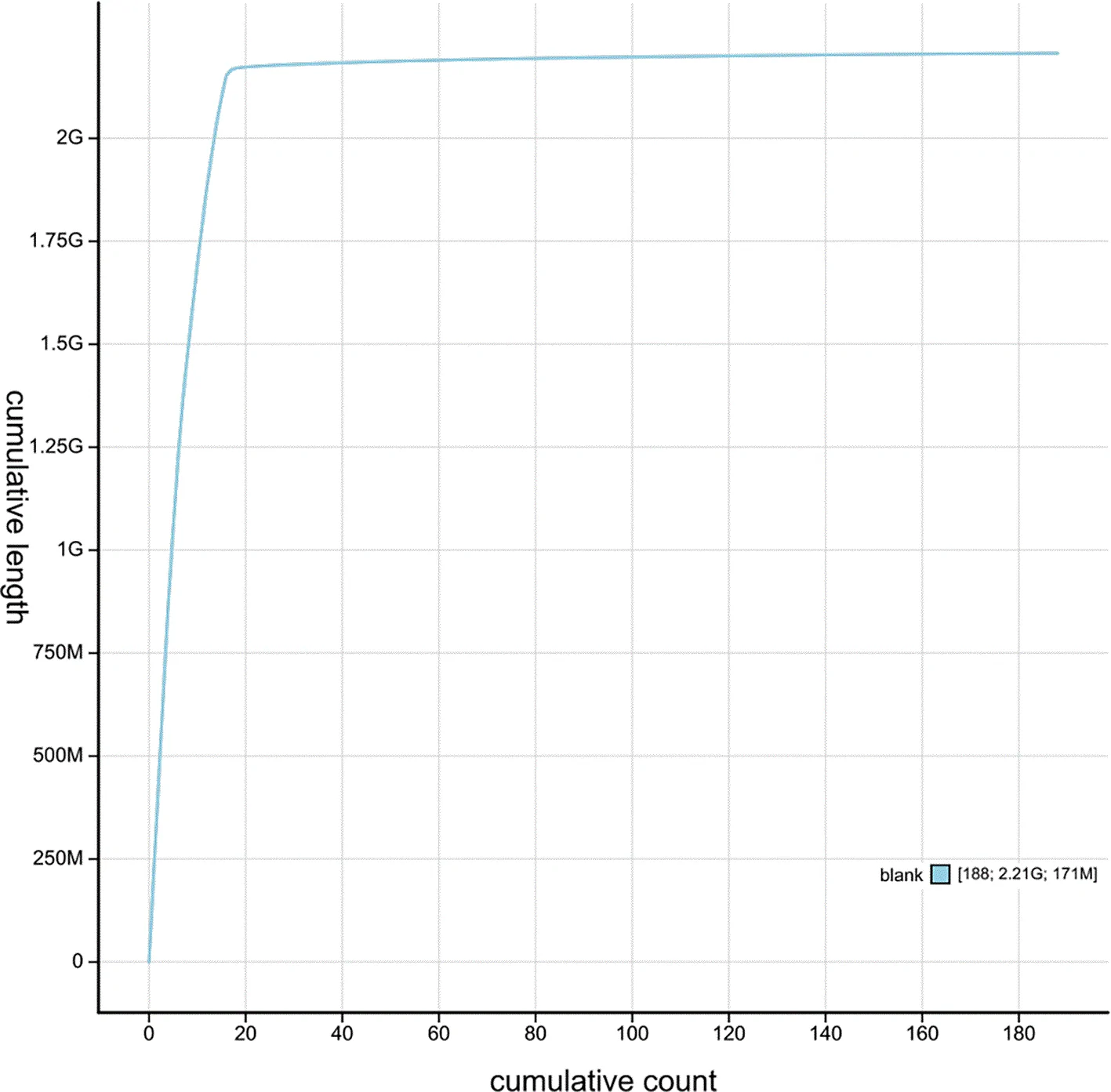
Cumulative sequence plot generated for the Gardnerycteris keenani assembly using BlobToolKit. The grey line shows the cumulative length for all chromosomes/scaffolds in the assembly. Colored lines represent Phylum represented in the assembly.

**Table 3.** Software tools used.

Software tool	Version	Source
PretextGraph	v0.0.6	https://github.com/sanger-tol/PretextGraph
PretextMap	v0.1.9	https://github.com/sanger-tol/PretextMap/releases
MultiQC	1.11	https://github.com/ewels/MultiQC
Genomescope	2.0	https://github.com/tbenavi1/genomescope2.0
Hifiasm	0.19.3	https://github.com/chhylp123/hifiasm
BUSCO	5.3.2	https://busco.ezlab.org/
Merqury	1.3	https://github.com/marbl/merqury
gfastats	1.3.6	https://github.com/vgl-hub/gfastats
Arima-HiC Mapping Pipeline	-	https://github.com/ArimaGenomics/mapping_pipeline
YaHS	1.2a	https://github.com/c-zhou/yahs
samtools	1.9	https://www.htslib.org/
BlobToolKit	3.2.7	https://github.com/blobtoolkit/blobtoolkit
PretextView	1.0.0	https://github.com/sanger-tol/PretextView/releases/tag/1.0.0
BLAST	2.10.1	https://ftp.ncbi.nlm.nih.gov/blast/executables/blast+/2.10.1/
Kraken2	2.1.1	https://github.com/DerrickWood/kraken2
BWA-MEM2	2.2.1	https://github.com/bwa-mem2/bwa-mem2

## Ethics and consent

Capture and sampling were conducted under Belize Forest Department Permit FD/WL/1/21(16) and samples were exported under Belize Forest Department permit FD/WL/7/22(07). All work was conducted with approval by the AMNH Institutional Animal Care and Use Committee (AMNHIACUC-20210614 given on 6/14/2021). All efforts were made to ameliorate suffering of animals.

## Data Availability

The ***Gardnerycteris keenani*** genome sequencing initiative is part of the Bat1K genome sequencing project. The genome assembly is released openly for reuse. Underlying data may be available for non-commercial research purposes upon request. Please email bat1kconsortium@gmail.com for more information. The genome assembly can be found in the European Nucleotide Archive: mGarKee1.hap1. Accession number: GCA_050655435.1, https://www.ebi.ac.uk/ena/browser/view/GCA_050655435.1 NCBI BioProject: Gardnerycteris keenani isolate: mGarKee1. Accession number: PRJNA1270020, https://www.ncbi.nlm.nih.gov/datasets/genome/GCA_050655435.1/ under the Bat1K BioProject PRJNA489245. Data accession identifiers are reported in Table 1. Zenodo: The genome sequence of Gardnerycteris keenani (Chiroptera, Phyllostomidae, Stenodermatinae; Handley, 1960), https://doi.org/10.5281/zenodo.20456787 ( BAT1K, 2026). Data are available under the terms of the Creative Commons Attribution 4.0 International license (CC-BY 4.0).
