# Supplementary material for: The genome sequence of Gardnerycteris keenani (Chiroptera, Phyllostomidae, Stenodermatinae; Handley, 1960)
Source: Wellcome Open Res. 2026 Aug 18;11:527. [Version 1] doi: 10.12688/wellcomeopenres.26494.1 (PMC13562959; doi:10.12688/wellcomeopenres.26494.1)
Supplement: Peer review history

# Reviewer report: Reviewer response for version 1

**DOI:** 10.21956/wellcomeopenres.29175.r166389

**Published:** 2026-09-09

**Authors:** Xiuguang Mao

**Affiliations:** 1 East China Normal University, Shanghai, China

This manuscript presents a high-quality chromosome-scale genome assembly for *Gardnerycteris keenani*, following the standards of the Bat-1k sequencing project. For the current version of the manuscript, I have the following three comments:

 1) The authors state that "The majority of the assembly is scaffolded into 32 chromosomal pseudomolecules, with the X and Y sex chromosomes assembled." Here, "32" should read "17."

 2) In the main text, the authors state that "the assembly has a BUSCO completeness of 98.0% using the vertebrata_odb10 reference set." However, in Figure 4, the BUSCO database used was mammalia_odb10 (9,226 genes). Please ensure consistency in the database used and the reported BUSCO score.

 3) The authors state that "The quality of the assembly was evaluated using Merqury (Rhie et al., 2020) and BUSCO (Manni et al., 2021)." However, no QV (quality value) results for the genome assembly based on Merqury are reported. Please check this.

Are sufficient details of methods and materials provided to allow replication by others?

Yes

Is the rationale for creating the dataset(s) clearly described?

Yes

Are the datasets clearly presented in a useable and accessible format?

Yes

Are the protocols appropriate and is the work technically sound?

Yes

Reviewer Expertise:

Comparative genomics, genome assembly, evolutionary biology, speciation, bats

I confirm that I have read this submission and believe that I have an appropriate level of expertise to confirm that it is of an acceptable scientific standard.

# Reviewer report: Reviewer response for version 1

**DOI:** 10.21956/wellcomeopenres.29175.r165119

**Published:** 2026-08-27

**Authors:** Inge Seim

**Affiliations:** 1 Institute of Deep-Sea Science and Engineering, Sanya, China

In their Data Note, Simmons and colleagues report a high-quality genome assembly of a male *Gardnerycteris keenani* bat from Belize. They successfully assembled both sex chromosomes, although I am not in a position to evaluate their completeness relative to the best current bat assemblies. **I have no major concerns and recommend acceptance of this genome report after the authors address the minor issues outlined below**.

 • Table 1 lists multiple tissue types (muscle, liver, testes, spleen, kidney), but the Methods section only mentions that liver was used for HiFi and Hi-C sequencing. What were the other tissues used for? I assume RNA sequencing, but the manuscript provides no information. The NCBI BioSample entry (https://www.ncbi.nlm.nih.gov/biosample/?term=SAMN48711179) lists all tissues, which is confusing—it seems unlikely they were all pooled before sequencing…

 • Table 2: Consider using the same number of decimal places throughout (e.g., 43.0 instead of 43 for consistency with other values in Table 2).

 • Table 2 (and elsewhere): Some text is in bold. What is the rationale for this formatting? Please standardise.

 • Methods: The sentence "Primary assembly contigs were scaffolded with chromosome confirmation Hi-C data" is awkward and contains a spelling error (confirmation should be conformation, I think). I suggest: "The primary assembly contigs were scaffolded using high-throughput chromosome conformation capture (Hi-C) data."

 • Figures: Consider inverting the text labels on the *y*-axes for better readability.

 • Methods section: Some content appears to belong in the "Ethics and consent" section rather than in Methods. Please “relocate”/shift-around as appropriate.

 • Ethics and consent section: The phrase "All efforts were made to ameliorate suffering of animals" sounds unnecessary.

 • Figures, Table 6, Extended Data, and elsewhere: Please ensure that *Gardnerycteris keenani* is consistently italicised throughout. In most places, it would be clearer to use *G. keenani *after the first full mention too.

 • Finally (referring to the Latin name above), I realise that this manuscript may be part of a larger collection of Data Notes generated with the assistance of AI tools such as Claude, but I would still encourage the authors to proofread more carefully.

Are sufficient details of methods and materials provided to allow replication by others?

Yes

Is the rationale for creating the dataset(s) clearly described?

Yes

Are the datasets clearly presented in a useable and accessible format?

Partly

Are the protocols appropriate and is the work technically sound?

Yes

Reviewer Expertise:

comparative genomics, endocrinology, molecular evolution

I confirm that I have read this submission and believe that I have an appropriate level of expertise to confirm that it is of an acceptable scientific standard.
